# Modulation of Emotional Conflict Processing by High-Definition Transcranial Direct Current Stimulation (HD-TDCS)

**DOI:** 10.3389/fnbeh.2019.00224

**Published:** 2019-09-24

**Authors:** Maria Kuehne, Katarina Schmidt, Hans-Jochen Heinze, Tino Zaehle

**Affiliations:** ^1^Department of Neurology, Otto-von-Guericke-University Magdeburg, Magdeburg, Germany; ^2^Center for Behavioral Brain Sciences, Magdeburg, Germany

**Keywords:** emotional control, tDCS—transcranial direct current stimulation, HD-tDCS, DLPFC (dorsolateral prefrontal cortex), N170 amplitude

## Abstract

Cognitive control is characterized by selective attention to relevant stimuli while irrelevant, distracting stimuli are inhibited. While the classical color-word Stroop task was implemented to investigate the processes of cognitive control, a variant of it—the face-word Stroop task—allows for directly investigating processes of emotional conflict control. It is thought that the prefrontal cortex (PFC) is especially involved in processes of cognitive control, while the rostral cingulate is mainly associated with the resolution of emotional conflict. In recent years, the role of the dorsolateral PFC (DLPFC) during the performance of the classical Stroop was investigated by means of transcranial direct current stimulation (tDCS) with divergent results. However, investigations to the causal role of the DLPFC during emotional conflict processing are rare. For this purpose, we used a combined high-definition tDCS (HD-tDCS)/electroencephalogram (EEG) setting to investigate the impact of anodal stimulation of the left DLPFC on behavioral and electrophysiological responses during an emotional face-word Stroop task. In two separate sessions, participants (*n* = 18) received either sham or anodal HD-tdc stimulation while responding to the emotional expression of the face and ignoring the word. Our results show that anodal stimulation of the left DLPFC increases the behavioral interference effect, that is, the already decelerated reaction times (RTs) to incongruent trials further increase while RTs to congruent trials remain largely unaffected. Furthermore, the stimulation modulates brain response to emotional facial expressions during the face-word Stroop generally—independent of the valence of the emotional expression and the congruency of the combined face-word presentation, the N170 decreases during anodal stimulation. These results reveal that the left DLPFC has a causal role in emotional conflict processing during a face-word Stroop.

## Introduction

Cognitive control supports flexible, adaptive responses and complex goal-directed behavior. Also called executive control, this process includes selectively attending to relevant information while inhibiting irrelevant information from the environment as well as flexible adjustments in performance (Cohen, 2017). A classical paradigm assessing cognitive control processes is the color-word Stroop task (Stroop, 1935). The Stroop task has become a standard task to investigate mechanisms of selective attention and top-down control of behavior (MacLeod, 1992; Banich et al., 2001). In the classical version of this task, subjects have to name the ink colors of color words. Compared to naming the ink color of a corresponding written color word (congruent condition), naming the ink color of an incongruent color word (incongruent condition) results in an increase in reaction times (RTs). This effect of slowing in RT is known as the Stroop interference or Stroop effect (Stroop, 1935; MacLeod, 1991). One explanation for the occurrence of this interference effect relates to different stages of automatic processing. The relatively automatic and overlearned process of word reading competes with the less automatic and more controlled process of naming the ink color. Thus, in the incongruent condition it requires more cognitive control to actively inhibit the automatically processed, yet, task-irrelevant information (written word) and selectively attend to the task-relevant information (color of the word; MacLeod, 1991, 1992; Banich et al., 2001). The resulting conflict occurs on a stimulus level (activation of ink color representation conflicts with the activation of the representation corresponding to the semantically meaning of the word; Hock and Egeth, 1970), as well as on motor response level (selection of correct response to ink color conflicts with response to task irrelevant word; Posner and Snyder, 1975).

In the past, neuroimaging and electroencephalogram (EEG) studies defined a distributed neuronal network underlying cognitive control processes during performance of the Stroop task. In particular, two brain areas have been associated with the regulative and evaluative processes of cognitive control—the prefrontal cortex (PFC), especially the dorsolateral and ventrolateral part (Miller and Cohen, 2001) and the anterior cingulate cortex (ACC; Posner and DiGirolamo, 1998). Several studies demonstrated that prefrontal regions execute regulative control processes as maintaining task demands, top-down control, allocation of attention to task-relevant information, demand for control resources, prearrangement of inhibiting task-inappropriate response alternatives and adjustments in behavior (Banich et al., 2000; MacDonald et al., 2000; Egner, 2011). In contrast, the ACC has been mainly associated with evaluative control processes such as monitoring of processing conflicts during error and high conflict trials (Kerns et al., 2004). The interaction between the ACC and PFC emphasizes the dynamic process of cognitive control (MacDonald et al., 2000). It is thought that ACC and PFC form a feedback loop where the ACC evaluates and detects conflicts due to interference or mistakes and signals when adjustments in control is necessary to achieve goal-directed behavior by recruitment of PFC as control implementer (Botvinick et al., 2001, 2004; van Veen et al., 2001).

In everyday life, our ongoing behavior is particularly determined by emotionally salient stimuli (Nummenmaa et al., 2006). In such situations, emotional conflicts emerge from the interference of goal-relevant emotional stimuli with goal-irrelevant emotional stimuli, which normally needs to be suppressed through conflict control mechanisms to optimize goal-directed behavior (Miller, 2000; Carter and van Veen, 2007; Egner et al., 2007). Thus, one has to inhibit the emotional distractor and resolve the “conflict” of emotion (Etkin et al., 2006; Egner et al., 2008). To investigate this emotional conflict control empirically, a variation of the classical Stroop paradigm—the emotional face-word Stroop task—was developed. In this Stroop version, participants are required to indicate the emotional expressions of faces while ignoring emotional words superimposed on the faces. As in the classical version, words can constitute a congruent- (face and word describe same emotional expressions) or incongruent (face and word indicate different emotional expression) condition. Thus, conflict arises when the lexical word information is incongruent to the facial affective stimulus (i.e., the word happy written across a sad face) resulting in the Stroop interference effect (Etkin et al., 2006).

Insights into the underlying brain dynamics during the execution of the emotional Stroop paradigm are determined by fMRI measurements. These data assume that the dorsolateral PFC (DLPFC) and amygdale are associated with emotional conflict detection while the rostral ACC is related to conflict resolution by inhibiting amygdalar responses to emotional task-irrelevant stimuli (Etkin et al., 2006). As indicated by electrophysiological data, the process of emotional interference starts relatively early—with increased amplitude of the face-sensitive N170 component to incongruent compared to congruent stimuli when participants are asked to indicate emotional expression, while during word indication tasks congruent stimuli evoke enhanced N170 amplitude (Zhu et al., 2010). The N170 constitutes an event-related potential (ERP) of enhanced amplitudes to faces compared to non-facial stimuli in an interval of 130 and 200 ms (Itier and Taylor, 2004a). The neural origin of this component was determined in differing but partly simultaneously active face processing brain regions [e.g., lateral inferior occipital cortex and posterior fusiform gyrus (Rossion et al., 2003) and posterior superior temporal sulcus (Itier and Taylor, 2004b)]. Although several studies report N170 amplitude differences between emotional and neutral faces, there is no consensus whether the expression of its amplitude is sensitive to specific facial emotions like sad, happy or angry faces (for meta-analysis, see Hinojosa et al., 2015).

While in fMRI and EEG studies generally the association between brain activation and behavior are drawn on correlational inferences only, noninvasive brain stimulation (NIBS) methods provide the opportunity to directly modulate related brain regions and thereby investigate the role of this brain region in a causal way. Transcranial direct current stimulation (tDCS) is an established NIBS method to modulate cortical excitability. TDCS delivers low currents to the cortex area of interest resulting in the modulation of cortical excitability. The current flows between an active and a reference electrode through the skull to the brain tissue, thereby inducing diminutions or enhancements of cortical excitability (Nitsche et al., 2008). The direction of the tDCS-induced effect depends on the current polarity. Anodal tDCS typically has an excitatory effect while cathodal tDCS decreases the cortical excitability in the region under the electrode (Nitsche et al., 2008). The spatial specificity of this effect is especially important when considering the effectiveness and precision of stimulation and can be controlled by i.e., the size of electrodes. In conventional tDCS studies, rectangular electrodes with an area of 35 cm^2^ are used. While this method displays the standard design, it bears the disadvantage of relative low focal effectiveness. To improve the spatial preciseness, so-called high-definition (HD)-tDCS has been introduced recently (Datta et al., 2009). This stimulation design uses a 4 × 1 ring electrode protocol to modulate neuronal excitability (Datta et al., 2012; Kuo et al., 2013; Heimrath et al., 2015) and allows for the parallel assessment of EEG data.

In the present study, we took advantage of the high focal HD-tDCS design to investigate the role of the DLPFC in emotional conflict control. For this purpose, we measured the behavioral performance of participants during a face-word Stroop task and simultaneously recorded EEG while they underwent anodal HD-tDCS or sham stimulation. Based on the evidence mentioned above, we hypothesize that anodal tDCS will alter cortical excitability of the lDLPFC and in turn, modulates emotional control processing.

## Materials and Methods

### Participants

Eighteen healthy subjects participated in the present study (mean age 24.4 SD = ± 2.6; 10 female). To assess current depressive disorder they completed the Beck Depression Inventory-II (BDI-II; Hautzinger et al., 2006). Additionally, all participants affirmed to have no neurological or psychiatric disease and had normal or corrected-to-normal vision. Participants were stimulated twice on two separate sessions (with at least 5 and a maximum 7 days between)—receiving anodal stimulation at active and sham stimulation at the other session—while measuring their behavioral and electrophysiological performance during a face-word Stroop task. To exclude any stimulation order effect, the order of stimulation condition was pseudorandomized across subjects such that half of the participants started with sham and ended with an anodal stimulation session, while the other half received anodal on the first and sham on the second day. The order of stimulation sessions was predetermined by an odd-even-even-odd stimulation protocol. All participants were naïve to the stimulation conditions as well as the aim of the study and signed informed consent prior to the measurements. The local Ethical Committee of the University of Magdeburg approved the study.

### Stimuli

**Figure 1:**
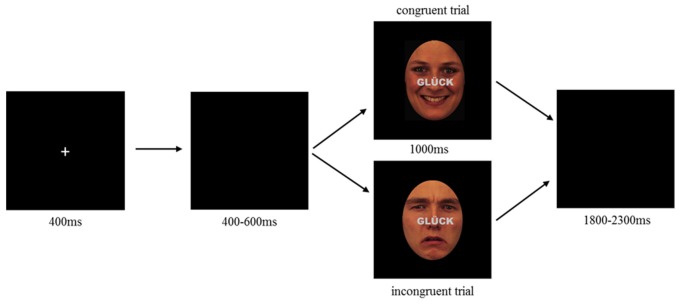
Emotional face-word Stroop paradigm illustrating an example of congruent (top) and incongruent (bottom) trial. Each trial started with the presentation of a fixation cross (400 ms) followed by a blank screen with a random interval between 400 and 600 ms. After blank, a combined emotional face and word was presented as congruent (top) or incongruent (bottom) stimulus lasting for 1,000 ms. Participants were requested to indicate whether the face displayed a neutral, happy or sad expression by pressing the corresponding button on a computer keyboard (buttons: V, B, N). Responses had to be given within 1,000 ms. Target buttons were pseudorandomly assigned to each participant, such that the possible allocation of response button and facial expression was balanced between participants. Each trial ended with a varying inter stimulus interval of 1,800–2,300 ms.

For the emotional face-word Stroop task, nine female and nine male characters were selected from Karolinska face data base (female: AF01, AF02, AF05, AF07, AF14, AF16, AF19, AF20, AF21; male: AM09, AM10, AM11, AM13, AM14, AM17, AM22, AM23, AM29; Lundqvist et al., 1998), each displayed happy, sad and neutral facial expressions resulting in 54 face stimuli. All stimuli were equally sized and oval shaped masked to exclude details like hairstyle (see Figure 1). Stimuli were further edited by inserting a written word across the face. Words comprised the German words for happiness, sadness and neutral (“GLÜCK,” “TRAUER,” “NEUTRAL”), centrally located between face and mouth region, printed in gray capitalized bold letters (see Figure 1). Stimuli were either presented congruently (emotion word corresponds to facial expression) or incongruently (emotion word contrasts facial expression), where for the incongruent condition happy faces were always contrasted with the word “sadness” and sad faces always with the word “happiness,” while neutral faces were always contrasted with “happiness.” Each emotional expression (3) of each character (18) is displayed in each congruency condition (2), resulting in 108 stimuli.

### HD-tDCS

**Figure 2:**
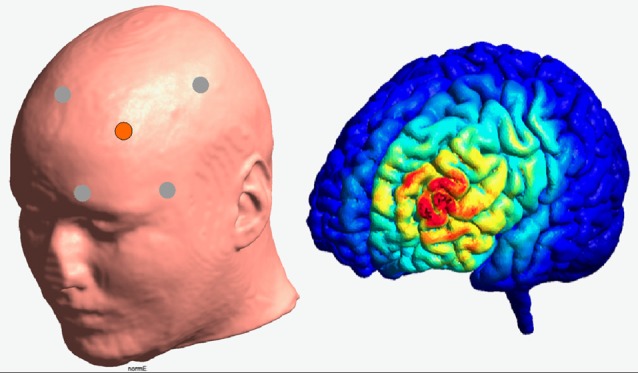
Illustration of the electrode positioning and modeled current density for the HD—stimulation (Jung et al., 2013).

Transcranial direct current stimulation was applied to the left DLPFC in a high-definition 4 × 1 ring configuration. For this purpose and according to the international 10–20 system, F3 electrode constituted the active electrode, surrounded by four reference electrodes (Fz, C3, FP1, F7). Brain modeling software (Jung et al., 2013) was used to ensure that this electrode placement is suitable to modulate the activity of the left DLPFC (see Figure 2). The sintered Ag/AgCl ring electrode (outer radius 12 mm, inner radius 6 mm) was fixed on a EEG cap and filled with EEG electrolyte gel (Easy Cap, Abralyt 2000) to improve the contact and thus the conductance between electrode and skin. Impedances were under 5 kΩ and were kept constant between electrodes. A battery-driven constant current stimulator (NeuroConn gmbH, Ilmenau, Germany) delivered the current with a strength of 0.5 mA with a linear fade in and fade out of 5 s. Stimulation started 10 min before the measurement to ensure stable stimulation effects in accordance with Nitsche et al. (2008) and ended with the termination of the experiment. In contrast to anodal session, the stimulation during the sham condition was applied for 30 s only. On the 2nd stimulation session, participants were asked to indicate whether and when they received active or sham stimulation.

### EEG Recordings

EEG was recorded from Ag/AgCl electrodes at positions F4, F8, Cz, C4, T7, T8, Pz, P3, P4, P7, P8, PO3, PO4, PO7, PO8, Oz and right mastoid according to 10–20 system. Horizontal and vertical electrooculogram (HEOG/VEOG) was measured from two electrodes placed below and lateral to the left eye. Impedances were kept below 5 kΩ. Data of all electrodes were referenced to left mastoid and digitally online filtered with a high pass filter of 0.1 Hz, recorded with Brainamp DC amplifier (Brainproducts) and corresponding recording software (BrainVision Recorder 1.20, Brain Products GmbH, Munich, Germany) at a sampling rate of 1,000 Hz.

### Procedure

To investigate the impact of HD-tDCS modulation of the lDPLFC on emotional conflict control, participants performed an emotional face-word Stroop during sham and anodal HD-tDCS. The experiment was conducted in a dimly lit room where participants sat in a comfortable chair with view orientation towards a display located in front of them. After EEG and HD-tDCS preparation, the experiment started with an initial HD-tDCS stimulation (either anodal or sham) of 10 min, thereafter the face-word Stroop and EEG recording started simultaneously to the ongoing stimulation. During the initial stimulation phase, participants performed a practice block to get familiar with the paradigm and response possibilities. During the face-word Stroop, participants had to indicate the emotional expression of the face while ignoring a written word across the face. Stimulus presentation was controlled by Presentation^®^ software (Version 19, Neurobehavioral System, Inc., Berkeley, CA, USA). The stimulation continued during the entire duration of the task where all 108 trials were presented within one block lasting approximately 7 min. Each trial started with the presentation of a fixation cross (400 ms) followed by a blank display with random presentation duration of 400–600 ms, hereafter combined face-word stimuli were presented for 1,000 ms. Each trial ended with a blank display with a random interval of 1,800–2,300 ms. Trials were randomly presented such that there were no restrictions regarding repetition condition (see Figure 1).

### EEG Data Analysis

To investigate the impact of HD-tDCS modulation of lDPLFC activity on electrophysiological level, the face sensitive N170 was assessed. For this purpose, EEG data were processed using Brain Vision Analyzer (version 2.1, Brain Products GmbH, Munich, Germany). Only trials with correct responses within a time window of 1,000 ms after stimulus onset were selected. In a first preprocessing step, data were bandpass filtered between 1 and 30 Hz using a 2nd order zero-phase IIR Butterworth filter (24 dB/oct) and segmented into 1,200 ms epochs (−200 ms prestimulus interval) relative to the onset of the face stimulus. Those epochs with artifacts were excluded from further analyses. The artifact rejection proceeded semiautomatic in accordance with pre-determined rejection criteria (maximal allowed voltage step of 50 μV/ms, maximal allowed difference of values in intervals 200 μV, lowest allowed activity in intervals of 0.5 μV). Following this artifact rejection procedure, on average 3.78 trials (SD ± 3.50) were excluded in sham condition and 6.44 trials (±4.40 SD) in anodal stimulation condition. Subsequently, artifact-free data were averaged separately for *congruency* and *valence* (i.e., happy congruent, sad congruent, neutral congruent, happy incongruent, sad incongruent, neutral incongruent) for both stimulation sessions. Based on previous research (Zhu et al., 2010; Eimer, 2011) and by visual inspection of grand-average waveforms, data of P7, PO7, P8 and PO8 were pooled. Peak detection for most negative deflection within a time window between 150 and 250 ms was conducted and subsequently mean amplitude values within a time window of 20 ms around the peak were extracted separately for each participant and each stimulus condition.

### Statistical Analysis

Statistical analysis for both, behavioral data as well as EEG data was performed using IBM SPSS software 24. Greenhouse-Geisser adjustment was applied for violations of sphericity. Finally, *post hoc* paired *t*-tests were conducted to further explore significant main or interaction effects.

### Behavioral Data

Responses faster than 200 ms and responses exceeding 1,000 ms were excluded from further analysis (sham stimulation: *M* = 6.5, SD ± 5.97, anodal stimulation: *M* = 8.56, SD ± 9.04). Further, incorrect responses were not included into the following statistical analysis (sham stimulation: *M* = 3.11, SD ± 1.63, anodal stimulation: *M* = 4.56, SD ± 2.99). Subsequently, two separate repeated-measures ANOVAs for the RTs and arcsine transformed error rates (ER) with the within-subject factors *stimulation* (anodal, sham), *valence* of the facial emotional expression (happy, sad, neutral) and *congruency* between target face and word (congruent, incongruent) were performed.

### EEG Data

Analogously, missing (no response between 200 and 1,000 ms) as well as incorrect responses were excluded from statistical analysis of EEG data. Mean amplitudes of the N170 were entered into a repeated-measures ANOVA with the within-subject factors *stimulation* (sham, anodal), *valence* of the facial emotional expression (happy, sad, neutral) and *congruency* between face and word (congruent, incongruent).

## Results

### Behavioral Performance

**Figure 3:**
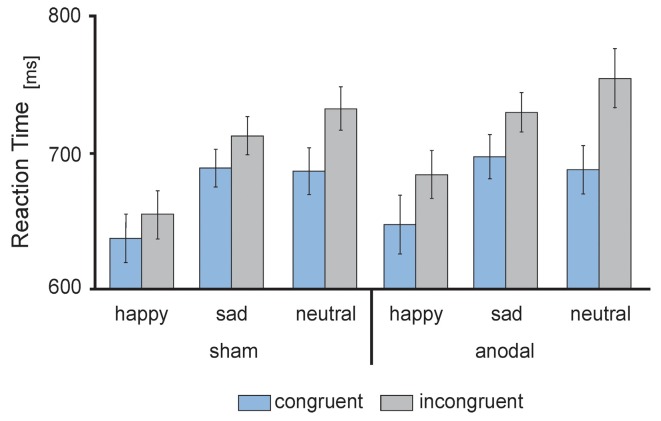
Behavioral performance: mean reaction times (RT) in ms, separately for sham (left) and anodal (right) stimulation for happy, sad and neutral faces during congruent (blue) and incongruent (gray) trials. Error bars represent SEM.

**Figure 4:**
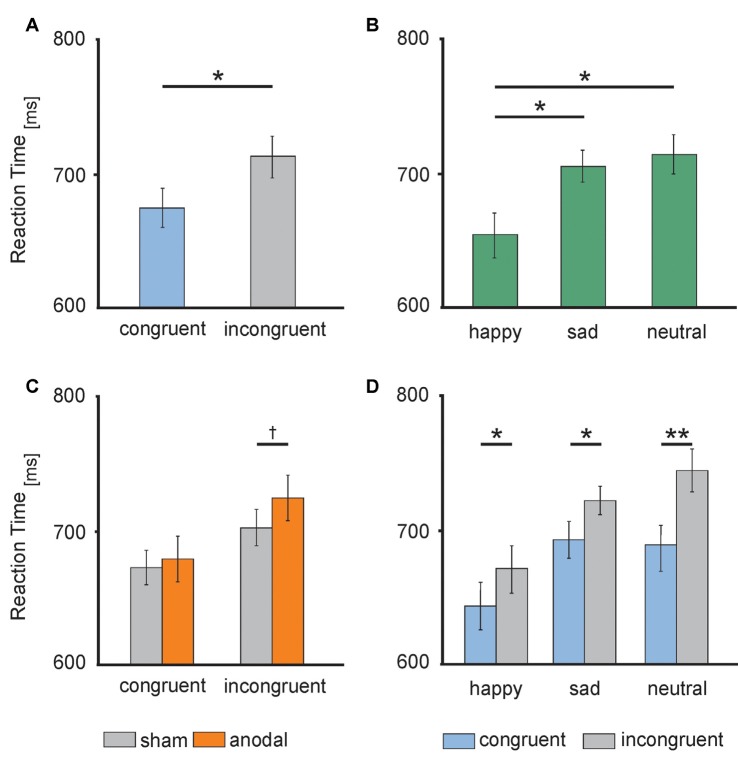
Behavioral performance: **(A)** RTs during congruent (blue) and incongruent (gray) trials. **(B)** RTs to happy (left), sad (middle) and neutral (right) faces. **(C)** RTs during anodal (orange) and sham (gray) stimulation separately for congruent (left) and incongruent (right) trials. **(D)** RTs to happy (left), sad (middle) and neutral (right) faces separately for congruent (blue) and incongruent (gray) trials. Error bars represent SEM. ^†^*p* ≤ 0.06, *p < 0.05, **p < 0.001.

RT data are presented in Figures 3, 4. The 2 × 3 × 2 repeated measures ANOVA revealed a significant main effect of the factor *congruency* (*F*_(1,17)_ = 37.559, *P* = 0.000) due to faster responses to congruent stimuli (*M* = 674.91 ms, SE = 13.74) compared to incongruent stimuli (*M* = 711.98 ms, SE = 14.51 ms, *t*_(17)_ = −6.129, *P* = 0.000; see Figure 4A) and *valence* of facial emotional expression (*F*_(2,34)_ = 27.859, *P* = 0.000) due to faster responses to happy faces (*M* = 656.47 ms, SE = 16.58) compared to sad (*M* = 707.83 ms, SE = 11.70, *t*_(17)_ = −5.744, *P* = 0.000) and neutral faces (*M* = 716.04 ms, SE = 15.30, *t*_(17)_ = −7.158, *P* = 0.000; see Figure 4B).

Furthermore, ANOVA revealed a significant interaction between the factors *stimulation* and *congruency* (*F*_(1,17)_ = 4.832, *P* = 0.042). This interaction was driven by a trend for increased RTs for incongruent stimuli during anodal tDCS (*M* = 723.05 ms, SE = 17.04) compared to sham stimulation (*M* = 700.90 ms, SE = 13.87, *t*_(17)_ = −1.992, *P* = 0.063; see Figure 4C). Finally, the ANOVA revealed a significant interaction between the factors *congruency* and *valence* of facial emotional expression (*F*_(2,34)_ = 8.726, *P* = 0.001) due to a more pronounced congruency effect for neutral faces [neutral congruent *M* = 687.95 ms, SE = 14.71, neutral incongruent *M* = 744.12 ms, SE = 16.72, *t*_(17)_ = −7.545, *P* = 0.000)] than for happy (happy congruent *M* = 642.84 ms, SE = 16.42, happy incongruent *M* = 670.09 ms, SE = 17.47, *t*_(17)_ = −3.892, *P* = 0.001) and sad faces (sad congruent *M* = 693.95 ms, SE = 13.14, sad incongruent *M* = 721.72 ms, SE = 11.63, *t*_(17)_ = −3.366, *P* = 0.004; see Figure 4D). There was no main effect of *stimulation* (*F*_(1,17)_ = 1.308, *P* = 0.269). Further, the interaction between *stimulation* and *emotion* (*F*_(2,34)_ = 0.15, *P* = 0.859) as well as between *stimulation* and *valence* and *congruence* (*F*_(2,34)_ = 0.179, *P* = 0.837) did not reach significance.

The 2 × 3 × 2 repeated measures ANOVA on ER revealed a significant main effect of the factor *congruency* (*F*_(1,17)_ = 7.848, *P* = 0.012) due to fewer errors to congruent stimuli (*M* = 0.094, SE = 0.012) compared to incongruent stimuli (*M* = 0.143, SE = 0.016, *t*_(17)_ = −2.802, *P* = 0.012) and *valence* of facial emotional expression (*F*_(2,34)_ = 3.639, *P* = 0.037) due to fewer errors to happy faces (*M* = 0.067, SE = 0.02) compared to sad (*M* = 0.130, SE = 0.025, *t*_(17)_ = −1.849, *P* = 0.082) and neutral faces (*M* = 0.158, SE = 0.023, *t*_(17)_ = −2.546, *P* = 0.021). Furthermore, ANOVA revealed a significant *valence* × *congruency* interaction (*F*_(2,34)_ = 4.069, *P* = 0.026) due to more pronounced congruency effects for neutral (neutral congruent *M* = 0.118, SE = 0.029, neutral incongruent *M* = 0.198, SE = 0.027, *t*_(17)_ = −2.570, *P* = 0.020) and for happy faces (happy congruent *M* = 0.025, SE = 0.014, happy incongruent *M* = 0.111, SE = 0.034, *t*_(17)_ = −2.593, *P* = 0.019) than for sad faces (sad congruent *M* = 0.14, SE = 0.026, sad incongruent *M* = 0.121, SE = 0.029, *t*_(17)_ = 0.798, *P* = 0.436). There was no main effect of stimulation (*F*_(1,17)_ = 2.647, *P* = 0.122). Further, the interaction between *stimulation* and *emotion* (*F*_(2,34)_ = 0.446, *P* = 0.644), *stimulation* and *congruency* (*F*_(1,17)_ = 0.024, *P* = 0.877) as well as between *stimulation* and *valence* and *congruence* (*F*_(2,34)_ = 0.279, *P* = 0.758) did not reach significance.

### EEG Data

**Figure 5:**
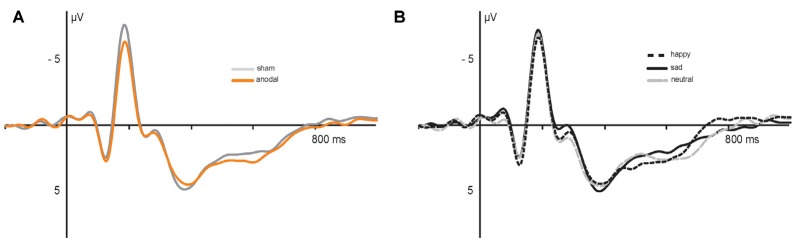
Electrophysiological data: grand average event-related potentials (ERPs) recorded during **(A)** anodal (orange) and sham (gray) stimulation and **(B)** in response to happy (dashed), sad (solid) and neutral (gray) faces.

**Figure 6:**
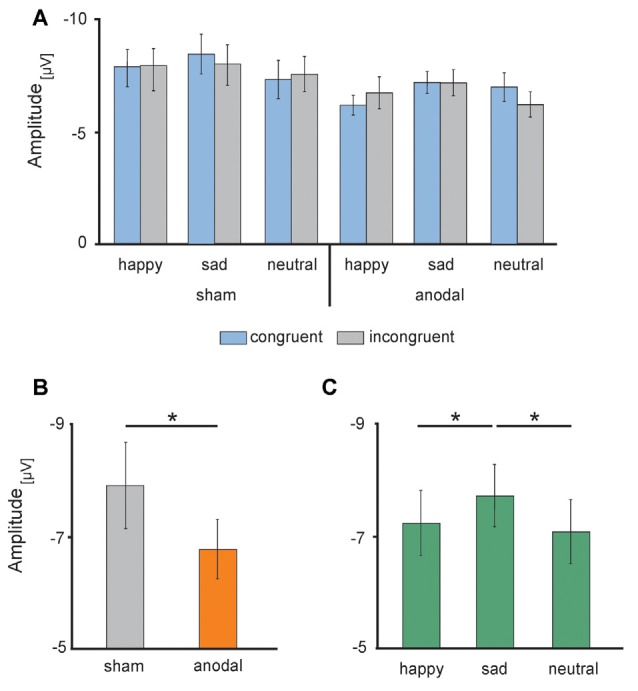
Electrophysiological data: high-definition transcranial direct current stimulation (HD-tDCS) induced changes in the N170 amplitudes **(A)** separately for sham (left) and anodal (right) stimulation for happy, sad and neutral faces during congruent (blue) and incongruent (gray) trials, **(B)** during anodal (orange) and sham (gray) stimulation and **(C)** in response to happy (left), sad (middle) and neutral (right) faces. Error bars represent SEM. *p < 0.05.

Electrophysiological data are presented in Figures 5, 6. The 2 × 3 × 2 repeated measures ANOVA for the N170 amplitude revealed a significant main effect of the factor *stimulation* (*F*_(1,17)_ = 6.131, *P* = 0.024) due to significant decreased N170 amplitude during anodal stimulation (*M* = −6.81 μV, SE = 0.53) compared to sham stimulation (*M* = −7.93 μV, SE = 0.78, *t*_(17)_ = −2.476, *P* = 0.024; see Figure 6B). Further, ANOVA revealed a significant main effect of the factor *valence*, due to highest N170 amplitude for sad faces (*M* = −7.77 μV, SE = 0.66) compared to happy faces (*M* = −7.24 μV, SE = 0.63, *t*_(17)_ = 2.659, *P* = 0.017) and neutral faces (*M* = −7.12 μV, SE = 0.63, *t*_(17)_ = −3.153, *P* = 0.006; see Figure 6C). The ANOVA revealed no significant main effect of *congruency* (*F*_(1,17)_ = 0.283, *P* = 0.601) as well as no significant interaction effects (*stimulation* × *emotion*: *F*_(2,34)_ = 1.024, *P* = 0.370; *stimulation* × *congruency*: *F*_(1,17)_ = 0.00, *P* = 0.985; *emotion* × *congruency*: *F*_(2,34)_ = 1.061, *P* = 0.357, *stimulation* × *emotion* × *congruency*: *F*_(2,34)_ = 2.14, *P* = 0.133).

## Discussion

The present study investigates the impact of HD-tDCS on emotional conflict processing. For this purpose, we applied anodal HD-tDCS over the left DLPFC while participants performed an emotional face-word Stroop task and simultaneously measured behavioral and electrophysiological responses. To our knowledge, this is the first study modulating the activity of left DLPFC by means of HD-tDCS while simultaneously recording EEG data during cognitive control.

In result, we show that behaviorally, the face-word Stroop task induced a general interference effect that was additionally modulated by the valence of the processed faces. Importantly, HD-tDCS modulated this interference effect. Under tDCS, participants tended to slow in response times during incongruent trials only, while performance of congruent trials remained unaffected. Finally, the direct electrophysiological data revealed a general effect of the DLPFC stimulation. HD-tDCS consistently decreased the amplitude of the N170 ERPs.

Despite the novel results reported in this study, there are some limitations that have to be acknowledged. First, since this is the first study investigating the influence of HD-tDCS of the lDLPFC on behavioral and electrophysiological data measured during a face-word Stroop task with a rather small sample, further studies are needed to make reliable conclusion. Second, as the performance during a Stroop task depends on attention as well as facial expression discriminations skills, future studies should additionally measure the individual level of the related capabilities to consider results in a more differentiated way and exclude participants with deficits related to these skills. Third, the present study did not control for sequence effects of congruent and incongruent trials, while previous studies (e.g., Botvinick et al., 1999; Kerns et al., 2004; Egner, 2007) revealed a reduction in RTs of trials preceded by high-conflict trials compared to low-conflict trials (Gratton effect, Gratton et al., 1992). In accordance with the conflict-monitoring hypothesis (Botvinick et al., 2001) and with respect to the performance during the Stroop task, the dorsal part of the ACC detects the conflict signal during incongruent trials. In return, this conflict signal triggers adjustment in cognitive control implemented by the PFC especially the dorsolateral part of it (i.e., Botvinick et al., 2004; Kerns et al., 2004). This is an important issue for future research, which could concentrate on the influences of those sequence effects while modulating DLPFC activity by means of tDCS.

Generally, RT as well as ER data of the present study replicate the classical interference effect while performing a Stroop task—incongruent trials lead to longer RTs and higher ER compared to congruent trials. This interference effect is widely proven in classical Stroop paradigms (i.e., Stroop, 1935; Vendrell et al., 1995; Liotti et al., 2000) as well as in the face-word Stroop task (i.e., Etkin et al., 2006; Zhu et al., 2010; Shen et al., 2013; Xue et al., 2015, 2016) and results from a response competition between the distracting but automatically processed word stimulus and the target stimulus (facial valence in the present study, ink color in the classical Stroop task). Additionally, our data reveal a general advantage for the processing of happy faces, independent of congruency and stimulation session. This advantage is demonstrated by faster RTs to happy faces compared to sad and neutral faces. Such valence dependencies (i.e., happy face vs. sad face) were less addressed in previous literature. However, an advantage of positive stimuli has been shown in a study by Chechko et al. (2012) where responses to happy faces were faster than to sad and fearful faces, indicating a general processing advantage for positive facial expressions. However, since for incongruent trials neutral as well as sad face stimuli were always combined with the word *happy*, it cannot be completely excluded that this overrepresentation of the word happy further influenced the RTs to happy faces. Nonetheless, this face-word combination of incongruent trials did not influence the stimulation effect, which is consistent across the different valences of facial emotional expressions. Our data show that anodal HD-tDCS over the left DPLFC interacted with the behavioral congruency effect. The effect, however, was limited to incongruent trials. Interestingly, while data in our study indicate a slowing of response times for incongruent trials during anodal stimulation, former studies also reported opposing effects. In particular, two previous studies investigating the influence of conventional anodal tDCS over the left DLPFC on the performance in a classical Stroop (Jeon and Han, 2012) and a modified color-word Stroop task (Loftus et al., 2015). Jeon and Han (2012) demonstrated a general speeding during word naming condition and during the interference condition of the Stroop after 20 min of 1 mA conventional anodal tDCS over F3, corresponding to the left DLPFC. Using 2 mA anodal tDCS for 10 min over the left DLPFC, Loftus et al. (2015) also reported decreased RTs for incongruent trials after tDCS. While already the applied Stroop tasks differ between these studies and the present, both former investigations applied a pre-post design, assessing the effects of conventional tDCS from pre- to post-stimulation. While such repetitive testing might add an additional parameter, also the assessed tDCS influences can differ between online and offline effects (Martin et al., 2014).

Two recent studies investigated the effect of anodal HD-tDCS of the left (Gbadeyan et al., 2016) and right (Gbadeyan et al., 2016, 2019) DLPFC during a visual flanker task. In contrast to our study, cognitive control was enhanced after anodal HD-tDCS. However, considerable methodological differences between these and the current study do not allow for a direct comparison. Particularly, both former studies used a concentric HD-tDCS setup, where the smaller anode was placed in the ring center of a bigger cathode, while we used a 4 × 1 ring electrode placement to stimulate the DLPFC. Additionally, both former studies applied 1 mA while in the present study we used 0.5 mA only. Since current intensity (Hoy et al., 2013; Papazova et al., 2018), as well as stimulation setup, can have a strong impact on tDCS-effects, further systematic investigations are needed to assess these effects in more detail.

While previous research consistently associates the DLPFC with cognitive control processing, divergent assumptions on the implementation of these control processes exist. While some authors assume that the DLPFC solves conflicts by suppressing the processing of task-irrelevant information (i.e., Banich et al., 2019) other affirm that the DLPFC amplifies the processing of task-relevant information (i.e., Egner and Hirsch, 2005). In the present study, modulating the activity of the DLPFC by anodal tDCS increased RTs to incongruent trials and additionally decreased the face selective N170 component, independent of congruency and emotional valence.

It might be assumed that the reduced N170 amplitude represents a reduced processing of task-relevant faces and that, in turn, the stimulation amplifies the processing of the task-irrelevant word by distracting attention from the relevant face stimulus towards the irrelevant word stimulus. While during the congruent condition this enhanced automatic processing of the congruent word would support emotional face identification with no changes in RTs, the enhanced processing of the incongruent word would result in deterioration of task performance during incongruent trials. However, assuming that anodal stimulation results in excitation of the underlying brain region, the present results would contradict results of Banich et al. (2019). In this study, increased DLPFC activity was associated with decreased perceptual processing of task-irrelevant stimuli. An explanation of the discrepancy of results could rely on task demands; while in the present study participants were asked to indicate emotional valence of the face, they had to indicate the emotional category of the word in the study by Banich et al. (2019).

Finally, when applying tDCS as a tool to investigate brain mechanisms during cognitive processes one has to consider that the general assumption of the dichotic anodal/cathodal effect on brain activity should not be regarded as ultimate. This note for caution is supported by the fact that anodal stimulation does not necessarily result in an excitation. In contrast to the classical anodal excitation–cathodal inhibition theory, recent research demonstrate opposing effects with decreased reactivity after anodal stimulation (Chen et al., 2014) and increased reactivity of brain regions after cathodal stimulation (Zaehle et al., 2011). Furthermore, starting from an optimal level of brain performance in unstimulated condition, anodal/cathodal stimulation does not necessarily result in increase/decrease of the neuronal reactivity of the underlying brain region but may impair processing of it (Baldi and Bucherelli, 2005). Findings of recent studies support this assumption, that the conventional anodal excitation–cathodal inhibition polarity hypothesis cannot be regarded as representative for all tDCS modulation effects (for a review, see Jacobson et al., 2012). In a previous study, cathodal HD-tDC stimulation of the dorsal ACC results in faster responses while participants performed an emotional counting Stroop task (To et al., 2018). Additionally, slowing in RT during a working memory task was reported by Marshall et al. (2005) after anodal and cathodal stimulation of the DLPFC.

Our data show that anodal HD-tDC stimulation over the left DLPFC modulates brain response to facial expressions of emotions and increases the interference effect during a face-word Stroop task. However, from these results, it cannot be reliably concluded that anodal stimulation of left DLPFC influences cognitive control processes by modulating processing of task-relevant-, or by modulating task-irrelevant stimuli by amplifying or suppressing their processing. Furthermore, in contrast to the assumption that anodal stimulation generally results in excitatory effects of the underlying brain region and consequently enhanced cognitive processing, present results suggest that our stimulation setup disturbed the (optimal) DLPFC performance during cognitive control. For this, future studies are necessary to investigate whether HD-tDC stimulation of the DLPFC alters performance during the face-word Stroop task by modulating processing of the task-relevant face or task-irrelevant word.

## Data Availability Statement

The datasets generated for this study are available on request to the corresponding author.

## Ethics Statement

The studies involving human participants were reviewed and approved by Local ethical committee of the University of Magdeburg, Germany. The patients/participants provided their written informed consent to participate in this study.

## Author Contributions

MK and TZ designed the experiment, interpreted the data and wrote the main manuscript. MK and KS performed the experiment, recorded and analyzed the data. H-JH and TZ contributed to the conception of the study. H-JH contributed reagents, materials and analysis tools. All authors reviewed the manuscript, approved the final manuscript and agreed to be accountable for all aspects of the work. All authors read and approved the final manuscript.

## Conflict of Interest

The authors declare that the research was conducted in the absence of any commercial or financial relationships that could be construed as a potential conflict of interest.

## References

[B1] Baldi E. Bucherelli C. (2005). The inverted “u-shaped” dose-effect relationships in learning and memory: modulation of arousal and consolidation. Nonlinearity Biol. Toxicol. Med. 3, 9–21. 10.2201/nonlin.003.01.002 19330154 PMC2657842

[B2] Banich M. T. Milham M. P. Atchley R. A. Cohen N. J. Webb A. Wszalek T.. (2000). Prefrontal regions play a predominant role in imposing an attentional ‘set’: evidence from fMRI. Cogn. Brain Res. 10, 1–9. 10.1016/s0926-6410(00)00015-x 10978687

[B3] Banich M. T. Milham M. P. Jacobson B. L. Webb A. Wszalek T. Cohen N. J.. (2001). Attentional selection and the processing of task-irrelevant information: insights from fMRI examinations of the Stroop task. Prog. Brain Res. 134, 459–470. 10.1016/s0079-6123(01)34030-x 11702561

[B4] Banich M. T. Smolker H. R. Snyder H. R. Lewis-Peacock J. A. Godinez D. A. Wager T. D.. (2019). Turning down the heat: neural mechanisms of cognitive control for inhibiting task-irrelevant emotional information during adolescence. Neuropsychologia 125, 93–108. 10.1016/j.neuropsychologia.2018.12.006 30615898 PMC6771039

[B6] Botvinick M. M. Braver T. S. Barch D. M. Carter C. S. Cohen J. D. (2001). Conflict monitoring and cognitive control. Psychol. Rev. 108, 624–652. 10.1037/0033-295X.108.3.624 11488380

[B7] Botvinick M. M. Cohen J. D. Carter C. S. (2004). Conflict monitoring and anterior cingulate cortex: an update. Trends Cogn. Sci. 8, 539–546. 10.1016/j.tics.2004.10.003 15556023

[B5] Botvinick M. Nystrom L. E. Fissell K. Carter C. S. Cohen J. D. (1999). Conflict monitoring versus selection-for-action in anterior cingulate cortex. Nature 402, 179–181. 10.1038/46035 10647008

[B8] Carter C. S. van Veen V. (2007). Anterior cingulate cortex and conflict detection: an update of theory and data. Cogn. Affect. Behav. Neurosci. 7, 367–379. 10.3758/cabn.7.4.367 18189010

[B9] Chechko N. Kellermann T. Zvyagintsev M. Augustin M. Schneider F. Habel U. (2012). Brain circuitries involved in semantic interference by demands of emotional and non-emotional distractors. PLoS One 7:e38155. 10.1371/journal.pone.0038155 22666470 PMC3362560

[B10] Chen J. C. Hämmerer D. Strigaro G. Liou L. M. Tsai C. H. Rothwell J. C.. (2014). Domain-specific suppression of auditory mismatch negativity with transcranial direct current stimulation. Clin. Neurophysiol. 125, 585–592. 10.1016/j.clinph.2013.08.007 24051072

[B11] Cohen J. D. (2017). “Cognitive control,” in The Wiley Handbook of Cognitive Control, ed. T. Egner (Weinheim: John Wiley and Sons, Ltd.), 1–28.

[B100] Datta A. Bansal V. Diaz J. Patel J. Reato D. Bikson M. (2009). Gyri-precise head model of transcranial direct current stimulation: improved spatial focality using a ring electrode versus conventional rectangular pad. Brain stimulation 2, 201–207. 10.1016/j.brs.2009.03.005 20648973 PMC2790295

[B13] Datta A. Truong D. Minhas P. Parra L. C. Bikson M. (2012). Inter-individual variation during transcranial direct current stimulation and normalization of dose using MRI-derived computational models. Front. Psychiatry 3:91. 10.3389/fpsyt.2012.00091 23097644 PMC3477710

[B14] Egner T. (2007). Congruency sequence effects and cognitive control. Cogn. Affect. Behav. Neurosci. 7, 380–390. 10.3758/cabn.7.4.380 18189011

[B15] Egner T. (2011). Right ventrolateral prefrontal cortex mediates individual differences in conflict-driven cognitive control. J. Cogn. Neurosci. 23, 3903–3913. 10.1162/jocn_a_00064 21568631 PMC3641154

[B17] Egner T. Delano M. Hirsch J. (2007). Separate conflict-specific cognitive control mechanisms in the human brain. Neuroimage 35, 940–948. 10.1016/j.neuroimage.2006.11.061 17276088

[B18] Egner T. Etkin A. Gale S. Hirsch J. (2008). Dissociable neural systems resolve conflict from emotional versus nonemotional distracters. Cereb. Cortex 18, 1475–1484. 10.1093/cercor/bhm179 17940084

[B16] Egner T. Hirsch J. (2005). Cognitive control mechanisms resolve conflict through cortical amplification of task-relevant information. Nat. Neurosci. 8, 1784–1790. 10.1038/nn1594 16286928

[B19] Eimer M. (2011). The face-sensitivity of the n170 component. Front. Hum. Neurosci. 5:119. 10.3389/fnhum.2011.00119 22022313 PMC3196313

[B20] Etkin A. Egner T. Peraza D. M. Kandel E. R. Hirsch J. (2006). Resolving emotional conflict: a role for the rostral anterior cingulate cortex in modulating activity in the amygdala. Neuron 51, 871–882. 10.1016/j.neuron.2006.07.029 16982430

[B21] Gbadeyan O. McMahon K. Steinhauser M. Meinzer M. (2016). Stimulation of dorsolateral prefrontal cortex enhances adaptive cognitive control: a high-definition transcranial direct current stimulation study. J. Neurosci. 36, 12530–12536. 10.1523/JNEUROSCI.2450-16.2016 27974612 PMC6705663

[B22] Gbadeyan O. Steinhauser M. Hunold A. Martin A. K. Haueisen J. Meinzer M. (2019). Modulation of adaptive cognitive control by prefrontal high-definition transcranial direct current stimulation in older adults. J. Gerontol. B Psychol. Sci. Soc. Sci. [Epub ahead of print]. 10.1093/geronb/gbz048 31045231

[B23] Gratton G. Coles M. G. Donchin E. (1992). Optimizing the use of information: strategic control of activation of responses. J. Exp. Psychol. Gen. 121:480. 10.1037//0096-3445.121.4.480 1431740

[B24] Hautzinger M. Keller F. Kühner C. (2006). Beck Depressions-Inventar (BDI-II). Frankfurt: Harcourt Test Services.

[B25] Heimrath K. Breitling C. Krauel K. Heinze H. J. Zaehle T. (2015). Modulation of pre-attentive spectro-temporal feature processing in the human auditory system by HD-t DCS. Eur. J. Neurosci. 41, 1580–1586. 10.1111/ejn.12908 25847301

[B26] Hinojosa J. A. Mercado F. Carretié L. (2015). N170 sensitivity to facial expression: a meta-analysis. Neurosci. Biobehav. Rev. 55, 498–509. 10.1016/j.neubiorev.2015.06.002 26067902

[B27] Hock H. S. Egeth H. (1970). Verbal interference with encoding in a perceptual classification task. J. Exp. Psychol. 83, 299–303. 10.1037/h0028512 5480902

[B28] Hoy K. E. Emonson M. R. Arnold S. L. Thomson R. H. Daskalakis Z. J. Fitzgerald P. B. (2013). Testing the limits: investigating the effect of tDCS dose on working memory enhancement in healthy controls. Neuropsychologia 51, 1777–1784. 10.1016/j.neuropsychologia.2013.05.018 23751169

[B29] Itier R. J. Taylor M. J. (2004a). N170 or N1? Spatiotemporal differences between object and face processing using ERPs. Cereb. Cortex 14, 132–142. 10.1093/cercor/bhg111 14704210

[B30] Itier R. J. Taylor M. J. (2004b). Source analysis of the N170 to faces and objects. Neuroreport 15, 1261–1265. 10.1097/01.wnr.0000127827.73576.d8 15167545

[B31] Jacobson L. Koslowsky M. Lavidor M. (2012). tDCS polarity effects in motor and cognitive domains: a meta-analytical review. Exp. Brain Res. 216, 1–10. 10.1007/s00221-011-2891-9 21989847

[B32] Jeon S. Y. Han S. J. (2012). Improvement of the working memory and naming by transcranial direct current stimulation. Ann. Rehabil. Med. 36, 585–595. 10.5535/arm.2012.36.5.585 23185722 PMC3503933

[B33] Jung Y.-J. Kim J.-H. Im C.-H. (2013). COMETS: a MATLAB toolbox for simulating local electric fields generated by transcranial direct current stimulation (tDCS). Biomed. Eng. Lett. 3, 39–46. 10.1007/s13534-013-0087-x

[B34] Kerns J. G. Cohen J. D. MacDonald A. W. III. Cho R. Y. Stenger V. A. Carter C. S. (2004). Anterior cingulate conflict monitoring and adjustments in control. Science 303, 1023–1026. 10.1126/science.1089910 14963333

[B35] Kuo H. I. Bikson M. Datta A. Minhas P. Paulus W. Kuo M. F.. (2013). Comparing cortical plasticity induced by conventional and high-definition 4 x 1 ring tDCS: a neurophysiological study. Brain Stimul. 6, 644–648. 10.1016/j.brs.2012.09.010 23149292

[B36] Liotti M. Woldorff M. G. Perez R. Mayberg H. S. (2000). An ERP study of the temporal course of the Stroop color-word interference effect. Neuropsychologia 38, 701–711. 10.1016/s0028-3932(99)00106-2 10689046

[B37] Loftus A. M. Yalcin O. Baughman F. D. Vanman E. J. Hagger M. S. (2015). The impact of transcranial direct current stimulation on inhibitory control in young adults. Brain Behav. 5:e00332. 10.1002/brb3.332 25874165 PMC4389055

[B12] Lundqvist D. Flykt A. Öhmann A. (1998). The Karolinska Directed Emotional Faces—KDEF, CD ROM. Stockholm: Karolinska Institutet, Department of Clinical Neuroscience, Psychology Section.

[B38] MacDonald A. W. III. Cohen J. D. Stenger V. A. Carter C. S. (2000). Dissociating the role of the dorsolateral prefrontal and anterior cingulate cortex in cognitive control. Science 288, 1835–1838. 10.1126/science.288.5472.1835 10846167

[B39] MacLeod C. M. (1991). Half a century of research on the Stroop effect: an integrative review. Psychol. Bull. 109, 163–203. 10.1037/0033-2909.109.2.163 2034749

[B40] MacLeod C. M. (1992). The Stroop task: the “gold standard” of attentional measures. J. Exp. Psychol. Gen. 121, 12–14. 10.1037//0096-3445.121.1.12

[B41] Marshall L. Mölle M. Siebner H. R. Born J. (2005). Bifrontal transcranial direct current stimulation slows reaction time in a working memory task. BMC Neurosci. 6, 23–23. 10.1186/1471-2202-6-23 15819988 PMC1090588

[B42] Martin D. M. Liu R. Alonzo A. Green M. Loo C. K. (2014). Use of transcranial direct current stimulation (tDCS) to enhance cognitive training: effect of timing of stimulation. Exp. Brain Res. 232, 3345–3351. 10.1007/s00221-014-4022-x 24992897

[B43] Miller E. K. (2000). The prefrontal cortex and cognitive control. Nat. Rev. Neurosci. 1, 59–65. 10.1038/35036228 11252769

[B44] Miller E. K. Cohen J. D. (2001). An integrative theory of prefrontal cortex function. Annu. Rev. Neurosci. 24, 167–202. 10.1146/annurev.neuro.24.1.167 11283309

[B45] Nitsche M. A. Cohen L. G. Wassermann E. M. Priori A. Lang N. Antal A.. (2008). Transcranial direct current stimulation: state of the art 2008. Brain Stimul. 1, 206–223. 10.1016/j.brs.2008.06.004 20633386

[B46] Nummenmaa L. Hyönä J. Calvo M. G. (2006). Eye movement assessment of selective attentional capture by emotional pictures. Emotion 6, 257–268. 10.1037/1528-3542.6.2.257 16768558

[B47] Papazova I. Strube W. Becker B. Henning B. Schwippel T. Fallgatter A. J.. (2018). Improving working memory in schizophrenia: Effects of 1mA and 2mA transcranial direct current stimulation to the left DLPFC. Schizophr. Res. 202, 203–209. 10.1016/j.schres.2018.06.032 29954701

[B48] Posner M. I. DiGirolamo G. J. (1998). Executive attention: conflict, target detection, and cognitive control. The Attentive Brain, ed. Parasuraman R. (Cambridge, MA: The MIT Press), 401–423.

[B49] Posner M. I. Snyder C. R. R. (1975). “Attention and cognitive control,” in Information Processing and Cognition: The Loyola Symposium, ed. Solso R. L. (Hillsdale, NJ: Lawrence Erlbaum), 55–85.

[B50] Rossion B. Joyce C. A. Cottrell G. W. Tarr M. J. (2003). Early lateralization and orientation tuning for face, word, and object processing in the visual cortex. Neuroimage 20, 1609–1624. 10.1016/j.neuroimage.2003.07.010 14642472

[B51] Shen Y. Xue S. Wang K. Qiu J. (2013). Neural time course of emotional conflict control: an ERP study. Neurosci. Lett. 541, 34–38. 10.1016/j.neulet.2013.02.032 23454616

[B52] Stroop J. R. (1935). Studies of interference in serial verbal reactions. J. Exp. Psychol. 18, 643–662. 10.1037/h0054651

[B53] To W. T. Eroh J. Hart J. Vanneste S. (2018). Exploring the effects of anodal and cathodal high definition transcranial direct current stimulation targeting the dorsal anterior cingulate cortex. Sci. Rep. 8:4454. 10.1038/s41598-018-22730-x 29535340 PMC5849683

[B54] van Veen V. Cohen J. D. Botvinick M. M. Stenger V. A. Carter C. S. (2001). Anterior cingulate cortex, conflict monitoring and levels of processing. Neuroimage 14, 1302–1308. 10.1006/nimg.2001.0923 11707086

[B55] Vendrell P. Junqué C. Pujol J. Jurado M. A. Molet J. Grafman J. (1995). The role of prefrontal regions in the Stroop task. Neuropsychologia 33, 341–352. 10.1016/0028-3932(94)00116-7 7792000

[B56] Xue S. Li Y. Kong X. He Q. Liu J. Qiu J. (2016). The dissociable neural dynamics of cognitive conflict and emotional conflict control: an ERP study. Neurosci. Lett. 619, 149–154. 10.1016/j.neulet.2016.03.020 26987720

[B57] Xue S. Ren G. Kong X. Liu J. Qiu J. (2015). Electrophysiological correlates related to the conflict adaptation effect in an emotional conflict task. Neurosci. Lett. 584, 219–223. 10.1016/j.neulet.2014.10.019 25459295

[B58] Zaehle T. Beretta M. Jancke L. Herrmann C. S. Sandmann P. (2011). Excitability changes induced in the human auditory cortex by transcranial direct current stimulation: direct electrophysiological evidence. Exp. Brain Res. 215, 135–140. 10.1007/s00221-011-2879-5 21964868

[B59] Zhu X.-R. Zhang H.-J. Wu T.-T. Luo W.-B. Luo Y.-J. (2010). Emotional conflict occurs at an early stage: evidence from the emotional face-word Stroop task. Neurosci. Lett. 478, 1–4. 10.1016/j.neulet.2010.04.036 20417689

